# 

**Published:** 1879-03

**Authors:** 


### Table of Contents.

### ORIGINAL COMMUNICATIONS.

THE HUMAN EYE. *By H. F. Scott, M.D., Atlanta, Ga.* .............. 705
FOREIGN BODIES IN EAR AND NOSE. *By A. Couvert, M.D., Oxford, Ga.* 714
VALEDICTORY ADDRESS TO THE GRADUATING CLASS IN ATLANTA MEDICAL COLLEGE. *By Col. J. S. Boynton, Griffin, Ga* ................ 716
VALEDICTORY ADDRESS OF DR. W. W. JAMES ...................... 724
DIFFICULT LABOR IN TWINS. *By J. Lovick Sappington, M.D., West Point, Ga* ........................................ 730

### REPORTS OF SOCIETIES.

Atlanta Academy of Medicine .................................. 731
Proceedings of the Boston Society for Medical Observation .......... 743
Proceedings of New Orleans Medical and Surgical Association ...... 746
Detroit Academy of Medicine .................................. 748

### SELECTIONS.

Note on Intra-Uterine Medication and Sterility .................... 751

### EDITORIAL.

Atlanta Medical College ...................................... 754
In Memoriam .................................................. 757
Editorial Correspondence ..................................... 758

### BIBLIOGRAPHICAL.

Index to Original Communications in the Medical Journals of the United States and Canada for 1877 .............................. 759
Diphtheria—Its Causes, Prevention and Proper Treatment .......... 760
New and Original Theories of the Great Physical Forces ........... 760
Pamphlets Received ........................................... 760

### EXCERPTA.

Damiana (Turnera Aphrodisiaca) ................................ 761
Opium Habit and Amyl Nitrite .................................. 761
Laceration of the Cervix a Cause of Sterility, Miscarriage, Etc. ....... 762
Skin Grafting en Masse ........................................ 762
Anomalous Twin Pregnancy ..................................... 763
How to Cough .................................................. 764

